# Cardiovascular magnetic resonance imaging patterns of acute COVID-19 mRNA vaccine-associated myocarditis in young male patients: A first single-center experience

**DOI:** 10.3389/fcvm.2022.965512

**Published:** 2022-08-23

**Authors:** Ruben Evertz, Alexander Schulz, Torben Lange, Sören J. Backhaus, Dirk Vollmann, Johannes T. Kowallick, Stephan von Haehling, Gerd Hasenfuß, Andreas Schuster

**Affiliations:** ^1^Department of Cardiology and Pneumology, University Medical Center Göttingen (UMG), Göttingen, Germany; ^2^German Center for Cardiovascular Research (DZHK), Göttingen, Germany; ^3^Herz- and Gefäßzentrum Göttingen, Göttingen, Germany; ^4^Institute for Diagnostic and Interventional Radiology, University Medical Center Göttingen (UMG), Göttingen, Germany

**Keywords:** COVID-19, myocarditis, mRNA-related, vaccination, cardiac magnetic resonance

## Abstract

**Background:**

The risk of myocarditis after mRNA vaccination against COVID-19 has emerged recently. Current evidence suggests that young male patients are predominantly affected. In the majority of the cases, only mild symptoms were observed. However, little is known about cardiac magnetic resonance (CMR) imaging patterns in mRNA-related myocarditis and their differences when compared to classical viral myocarditis in the acute phase of inflammation.

**Methods and results:**

In total, 10 mRNA vaccination-associated patients with myocarditis were retrospectively enrolled in this study and compared to 10 patients suffering from viral myocarditis, who were matched for age, sex, comorbidities, and laboratory markers. All patients (*n* = 20) were hospitalized and underwent a standardized clinical examination, as well as an echocardiography and a CMR. Both, clinical and imaging findings and, in particular, functional and volumetric CMR assessments, as well as detailed tissue characterization using late gadolinium enhancement and T1 + T2-weighted sequences, were compared between both groups. The median age of the overall cohort was 26 years (group 1: 25.5; group 2: 27.5; *p* = 0.57). All patients described chest pain as the leading reason for their initial presentation. CMR volumetric and functional parameters did not differ significantly between both groups. In all cases, the lateral left ventricular wall showed late gadolinium enhancement without significant differences in terms of the localization or in-depth tissue characterization (late gadolinium enhancement [LGE] enlargement: group 1: 5.4%; group 2: 6.5%; *p* = 0.14; T2 global/maximum value: group 1: 38.9/52 ms; group 2: 37.8/54.5 ms; *p* = 0.79 and *p* = 0.80).

**Conclusion:**

This study yielded the first evidence that COVID-19 mRNA vaccine-associated myocarditis does not show specific CMR patterns during the very acute stage in the most affected patient group of young male patients. The observed imaging markers were closely related to regular viral myocarditis in our cohort. Additionally, we could not find any markers implying adverse outcomes in this relatively little number of patients; however, this has to be confirmed by future studies that will include larger sample sizes.

## Introduction

The severe acute respiratory syndrome coronavirus 2 (SARS-CoV-2) pandemic has been a global challenge for the economic and medical systems. As of 22 May 2022, 525.4 million people have been infected by SARS-CoV-2 and more than 6 million people have died worldwide according to Johns Hopkins University (1, 2).

As a result of great efforts, 10 billion doses of newly developed COVID-19 vaccines have been administrated just within 2.3 years after the initial onset of the pandemic (1, 2). The European Medicine Agency (EMA) has authorized the use of five different vaccines, two of them are mRNA-based vaccines [Comirnaty by BioNTech and Spikevax (previously COVID-19 Vaccine Moderna) by Moderna], further two are the vector-based vaccines [Vaxzevria (previously COVID-19 Vaccine AstraZeneca) by AstraZeneca and Janssen by Johnson and Johnson], and one is protein based (Novaxovid by Novavax). While the advantages of the vaccination exceeded the potential side effects by far, the vaccine-associated myocarditis was called out as a threat and affecting young male patients, in particular (3, 4).

Myocarditis is defined as an injury of the heart muscle caused by inflammation in the absence of underlying ischemia (5). While the clinical presentation can be very heterogeneous and include unspecific symptoms, potential complications are associated with a poor outcome as myocarditis represents the major cause of cardiogenic shock in young adults (6, 7). Even though numerous myocarditis etiologies have been described, viral infections, such as SARS-CoV-2, are the most common ones (5). However, vaccinations for Smallpox and Influenza were previously also allocated with a potential risk to induce myocardial inflammation (8). This attributable risk of a vaccine-induced myocarditis is in accordance with the most recently described side effects of mRNA-based COVID-19 vaccinations (9–12) and was listed as a rare but potentially life-threatening side effect by the EMA and the USA Food and Drug Administration (FDA).

Current evidence suggests mRNA vaccination-related myocarditis as a condition that is predominantly affecting young male patients (9, 10–13), which usually occurs within days after the second vaccination dose (4–14).

While COVID-19-related myocarditis did not show major differences when compared to acute myocarditis of other causes, recent data demonstrated that particularly in COVID-19-related myocarditis, uncommon patterns of edema and late gadolinium enhancement (LGE) enhancement in contrast to COVID-19 vaccination-associated myocarditis were present (15, 16). However, COVID-19 vaccine-induced myocarditis on the other hand was compared with myocarditis of other causes and found to share clinical and imaging appearances in a heterogeneous cohort of different age and sex groups (17, 18). Consequently, this study is aimed to particularly discriminate patterns of acute COVID-19 vaccine-induced myocarditis in the primarily affected patient group of young male patients.

## Materials and methods

### Study population

In total, 20 male participants were retrospectively enrolled after an initial hospitalization due to one of the following diagnoses. Group 1: confirmed vaccination-associated myocarditis (between June and December 2021); group 2: non-vaccination-associated myocarditis (between September 2018 and October 2021). Myocarditis was considered as vaccination related to the cases within 2 weeks after COVID-19 vaccination and no other explanation was found, especially no other vaccination was given within the last month and no other symptoms of an infectious disease were present within 30 days prior to clinical presentation. Patients with classical viral myocarditis were primarily matched according to their age and sex in the first step. Secondly, patients within group 2 were matched with regard to cardiovascular risk factors and other comorbidities (cardiovascular risk factors and atrial fibrillation) of patients within group 1. For the remaining patients, we sought to balance laboratory markers as accurate as possible within both groups.

The initial referral of patients allocated to group 1 was for further evaluation of a suspected myocarditis due to COVID-19 vaccination or due to chest pain episodes with suspected myocardial ischemia during first diagnostic evaluations. Patients of group 2 were admitted with chest pain symptoms to our emergency department for ischemia rule-out. Diagnoses were based on clinical judgment, such as the clinical presentation, changes in electrocardiogram (ECG), and laboratory findings indicating myocardial damage. Furthermore, imaging findings had to be in accordance with the updated Lake Louise Criteria (19). The following criteria were defined as reasons for exclusion: (1) age < 18 years and >40 years, (2) an active SARS-CoV-2 infection at the time of the scan or within 4 weeks prior to the scan [detected by polymerase chain reaction (PCR) test], (3) other COVID-19 vaccination than mRNA-based ones, and (4) a history of coronary artery disease.

The study was approved by the local ethics committee. Due to the retrospective design of the study, the need for informed consent was waived.

### Diagnostic workup

Patients underwent a standardized clinical evaluation, including a detailed medical history, a physical examination, an ECG at rest, transthoracic echocardiography (TTE), cardiac magnetic resonance (CMR), and blood testing. ECG and TTE were performed in concordance to the ESC position paper for myocardial and pericardial diseases (20). TTE measurements included visual estimation of left ventricular ejection fraction (LVEF), tricuspid annular plane systolic excursion (TAPSE), and the visual assessment of the presence of pericardial effusion or wall motion abnormalities. Blood testing included high sensitive troponin T or I [due to the fact of different normal values, troponin levels are expressed as multiple times increment above the upper limit of normal (ULN)], creatine kinase (CK), creatine kinase muscle and brain (CK-MB), and c-reactive protein (CRP). Further diagnostic workup was based on the results of the latter tests.

### Cardiac magnetic resonance

Cardiac magnetic resonance was performed using a dedicated myocarditis protocol on a 3T Magnetom Vida (Siemens Healthcare GmbH, Erlangen, Germany) with a 32-channel anterior receiver coil in all patients. The protocol included a long- and short-axis stack of balanced steady state-free precession (bSSFP) slices with an in-plane resolution of 1.41 mm^3^ × 1.41 mm^3^ × 6 mm^3^ and a slice gap of 6 mm. LGE assessments were performed in phase-sensitive inversion recovery (PSIR) short-axis image stacks starting 15 min after injection of Gadobutrol (0.15 mmol/kg body weight) (Gadovist^®^, Bayer Healthcare, Berlin, Germany) (21). For quantitative T1 and T2 mapping, a single midventricular short-axis slice was obtained using a Modified Look-Locker Inversion Recovery (MOLLI) technique for T1 maps and a T2-Prep Fast Low Angle Shot (FLASH) at the same slice position for T2 maps, respectively (22, 23). Inline motion correction and the generation of pixel-based maps were automatically executed by the scanner.

### Image analysis

Postprocessing analyses were performed by an experienced observer blinded to all previously documented clinical information using commercially available Software (QMass^®^ and QStrain^®^, version 3.2.36.4, Medis Medical Imaging Systems, Leiden, Netherlands). Functional and volumetric parameters were assessed using semi-automated contouring detection with manual correction if necessary following established standards (24). Feature tracking strain analysis was based on three independently repeated measurements (25). Global longitudinal strain (GLS) was assessed from bSSFP image data in all three long-axis views (26, 27). The presence of LGE was visually evaluated by the reader followed by a quantification using the full-width half density method and was later displayed in absolute mass (grams) and its relation to the total left ventricular mass (percentage) (28, 29). T1 and T2 maps were screened for artifacts prior to analysis and affected segments were excluded from the analysis. During segmentation, the blood pool and right ventricular insertion point were carefully avoided. Furthermore, two specific regions of interest (ROIs) were defined as the septal region and the region with maximum values based on the color maps. Both ROIs were manually delineated. As suggested by the Society for Cardiovascular Magnetic Resonance (SCMR) and the European Association for Cardiovascular Imaging (EACVI), abnormal values were defined as T1 > 1,289 ms and T2 > 46 ms at the local facility (30). Extra cellular volume (ECV) was calculated as suggested by the SCMR with hematocrit obtained on the day before scanning. Abnormal values were defined as >30% at the local facility (30).

### Statistics

Statistical analysis was performed using IBM SPSS Statistics version 27 for Windows (International Business Machines Corporation (IBM^®^ Corp., Armonk, NY, United States). Continuous data were expressed as median ± interquartile range (IQR). Normal distribution for continuous data was tested using the Shapiro-Wilk test. In consequence, statistical significance was tested using Student’s *t*-test and the Mann-Whitney *U* test as appropriate. An alpha level of ≤0.05 was considered as statistically significant.

Intergroup comparison of categorical variables was performed using the χ^2^ test, and results were presented as absolute numbers and percentages. Nominal values were presented in percentages. Again, an alpha level of ≤0.05 was considered as statistically significant.

## Results

### Participant’s demographics

Patients’ characteristics are displayed in Table 1. Matching was performed successfully with a median age of 26.0 [21.3–32.8] years (group 1: 25.5 [21.8–33.5]; group 2: 27.5 [19.5–36.5]; *p* = 0.574). Cardiac risk factors and comorbidities were equally distributed across both groups (all *p* > 0.100). As by study design, predefined levels of troponin and creatinine kinase (CK, CK-MB) did not differ between both groups. The same was true for leucocytes and CRP (all *p* > 0.200; Table 2).

**TABLE 1:** Baseline characteristics.

Variable	All patients	Vaccine associated myocarditis	Non-vaccine associated myocarditis	*P*-value
Age (years)	26.00 (21.3–32.8)	25.50 (21.8–33.5)	27.5 (19.5–36.5)	0.574
Male [*n* (%)]	20 (100)	10 (100)	10 (100)	
Height (cm)	182 (176–187)	180 (174–187)	182 (179–188)	0.554
Weight (kg)	86 (68–93)	80 (67–90)	86 (68–97)	0.692
BMI (kg/m^2^)	24 (22–30)	24 (22–27)	24 (20–31)	0.740
**Comorbidities**				
Hypertension [*n* (%)]	1 (5)	1 (10)	0 (0)	0.305
Dyslipidaemia [*n* (%)]	1 (5)	0 (0)	1 (10)	0.305
Atrial fibrillation [*n* (%)]	2 (10)	1 (10)	1 (10)	1

Data are expressed as median (interquartile range), numbers, and percentage. Comparisons of vaccine-associated myocarditis and non-vaccine-associated myocarditis were performed. Continuous parameters were tested for normal distribution using the Shapiro-Wilk test and compared using the Mann-Whitney U test or t-test as appropriate. Categorical parameters were tested using a χ^2^ test. BMI, body mass index.

**TABLE 2:** Clinical presentation, blood test, and electrocardiogram (ECG) results at baseline.

Variable	All patients	Vaccine associated myocarditis	Non-vaccine associated myocarditis	*P*-value
**Symptoms at presentation**				
Chest pain [*n* (%)]	20 (100)	10 (100)	10 (100)	
Breathlessness [*n* (%)]	4 (20)	2 (20)	2 (20)	1
Palpitation [*n* (%)]	0 (0)	0 (0)	0 (0)	
**Blood tests**				
Troponin (x-fold above ULN)	92.9 (22.9–450.5)	125.3 (22.9–450.6)	92.90 (19.4–948.0)	0.418
CK (IU/l)	640.0 (248.5–829.5)	690.5 (508.25–886.50)	259.0 (120.0–745.0)	0.211
CK-MB (IU/l)	65.6 (31.78–90.5)	87.0 (58.6–95.5)	53.0 (23.5–99.8)	0.277
CRP (mg/l)	33.5 (6.8–65.2)	26.5 (13.9–45.5)	47.2 (4.9–101.0)	0.681
White blood cells (/μl)	9.4 (6.4–11.0)	8.2 (6.0–10.8)	9.5 (6.2–9.5)	0.499
**ECG results**				
ST-elevation [*n* (%)]	12 (60)	8 (80)	4 (40)	0.068
ST-depressions [*n* (%)]	1 (5)	1 (10)	0 (0)	0.279
T wave changes [*n* (%)]	4 (20)	2 (20)	2 (20)	1

Data are expressed as median (interquartile range), numbers, and percentage. Comparisons of vaccine-associated myocarditis and non-vaccine-associated myocarditis were performed. Continuous parameters were tested for normal distribution using the Shapiro-Wilk test and compared using the Mann-Whitney U test or t-test as appropriate. Categorical parameters were tested using a χ^2^ test. ULN, upper limit of normal; CK, creatine kinase; CK-MB, creatine kinase muscle and brain; CRP, c-reactive protein; ECG, electrocardiogram.

In group 1, all patients received mRNA vaccinations; with six of them vaccinated with Spikevax by Moderna and four patients with Comirnaty by BioNTech. All patients in both groups had chest discomfort as the main clinical symptom at the initial presentation (Table 2). Among the patients who received COVID-19 vaccination, two patients (20%) received the first dose and eight patients received (80%) the second. In group 2, myocarditis was consistently caused by non-COVID viral infections according to the medical records. The time between symptom onsets after vaccination in group 1 was 5.0 [3.5–7.3] days. CMR was performed promptly after symptom onset within 3.0 [1.0–5.5] days in group 1 and 2.0 [2.0–3.0] days in group 2, respectively (*p* = 0.239).

There was no clinical evidence of an underlying autoimmune disorder in any of the patients in group 1 or group 2.

### Electrocardiogram and transthoracic echocardiography

Electrocardiogram and TTE were obtained in all patients. The most prevalent ECG abnormality was ST elevation in 80% of group 1 and 40% of group 2. ST depression (group 1: 10%, group 2: 0%) and T wave changes (group 1: 20%, group 2: 20%) were less frequently present. No statistically significant differences between both groups could be observed (Table 2).

Left ventricular ejection fraction estimated by TTE was within the normal rage in most patients (group 1: LVEF 55% [50–55]; group 2: LVEF 55% [51.3–58.8] and without significant intergroup differences (*p* = 0.695). Right ventricular function measured by TAPSE was normal (above 16 mm) in all patients with no significant differences within both groups (*p* = 0.355). Furthermore, no statistically significant differences could be found for the presence of pericardial effusion (*p* = 0.136) or wall motion abnormalities (*p* = 0.329; Table 3).

**TABLE 3:** Echocardiographic characterization of the study population.

Variable	All patients	Vaccine associated myocarditis	Non-vaccine associated myocarditis	*P*-value
LVEF (%)	55 (51–55)	55 (50–55)	55 (51.3–58.8)	0.695
TAPSE (mm)	24.0 (20.2–29.4)	22.5 (19.6–27.3)	27.8 (20.6–29.9)	0.355
Wall motion abnormalities [*n* (%)]	6 (30)	4 (40)	2 (20)	0.329
Pericardial effusion [*n* (%)]	2 (10)	2 (20)	0 (0)	0.136

Data are presented as median (interquartile range), numbers and percentage. Comparisons of vaccine-associated myocarditis and non-vaccine-associated myocarditis were performed. Continuous parameters were tested for normal distribution using the Shapiro-Wilk test and compared using the Mann-Whitney U test or t-test as appropriate. Categorical parameters were tested using a χ^2^ test. LVEF, left ventricular ejection fraction; TAPSE, tricuspid annular plane systolic excursion.

### Cardiac magnetic resonance findings

Cardiac magnetic resonance results are presented in Tables 4, 5. Volumetric cardiac measurements for both ventricles were within normal range without any statistically significant differences (group 1: left ventricular end-diastolic volume index [LVEDVI] 91.0 ml/m^2^ [81.8–97.8], left ventricular end-systolic volume index [LVESVI] 40.0 ml/m^2^ [33.8–42.0]; group 2: LVEDVI 93.5 ml/m^2^ [78.8–99.5], LVESVI 38.0 ml/m^2^ [34.5–43.0]; *p* = 0.796 and *p* = 0.561). In addition, no differences were found in terms of functional measurements, e.g., left and right ventricular ejection fractions (group 1: LVEF 58.0% [52.0–64.5], RVEF 50.0% [46.8–53.3]; group 2: LVEF 58.0% [63.6–60.0], RVEF 54.0% [46.8–57.3]; *p* = 0.796 and *p* = 0.143). Furthermore, there were no differences in GLS (group 1: GLS −20.2 [−19.3 to −21.2]; group 2: GLS −20.4 [−18.2 to −22.5]; *p* = 0.912) on CMR.

**TABLE 4:** Cardiac magnetic resonance (CMR) volumetric results.

Variable	All patients	Vaccine associated myocarditis	Non-vaccine associated myocarditis	*P*-value
Time symptom to CMR (days)	2.0 (1.0–4.0)	3.0 (1.0–5.5)	2.0 (2.0–3.0)	0.239
**Left ventricle**				
LVMI (g/m^2^)	81.7 (68.5–94.4)	75.3 (56.5–100.8)	82.0 (81.5–89.6)	0.503
LVEDVI (ml/m^2^)	93.5 (80.0–98.5)	91.0 (81.8–97.8)	93.5 (78.8–99.5)	0.796
LVESVI (ml/m^2^)	39.5 (34.3–42.0)	40.0 (33.8–42.0)	38.0 (34.5–43.0)	0.561
LV-SVI (ml/m^2^)	53.5 (56.0–63.0)	58.0 (44.5–67.8)	53.0 (44.3–57.3)	0.436
LVEF (%)	58.0 (52.3–62.3)	58.0 (52.0–64.5)	58.0 (52.5–60.0)	0.796
GLS (%)	−20.2 (−21.9 to −18.6)	−20.2 (−21.2 to −19.3)	−20.4 (−22.5 to 18.2)	0.912
**Right ventricle**				
RVEDVI (ml/m^2^)	87.0 (78.5–94.0)	86.5 (69.8–94.0)	88.5 (81.5–99.8)	0.280
RVESVI (ml/m^2^)	43.0 (37.0–47.8)	45.0 (40.5–50.8)	39.5 (36.8–47.3)	0.315
RV-SVI (ml/m^2^)	47.0 (42.3–49.8)	46.5 (38.0–48.5)	47.5 (43.5–50.8)	0.660
RVEF (%)	52.0 (48.3–54.8)	50.0 (46.8–53.3)	54.0 (46.8–57.3)	0.143

Data are presented as median (interquartile range). Comparison of vaccination-associated myocarditis and non-vaccine-associated myocarditis was performed. Continuous parameters were tested for normal distribution using Shapiro-Wilk test and compared using the Mann-Whitney U test or t-test as appropriate. LVMI, left ventricular muscle index; LVEDVI, left ventricular end-diastolic volume index; LVESVI, left ventricular end-systolic volume index; LV-SVI, left ventricular stroke volume index; LVEF, left ventricular ejection fraction; GLS, global longitudinal strain; RVEDVI, right ventricular end-diastolic volume index; RVESVI, right ventricular end-systolic volume index; RV-SVI, right ventricular stroke volume index; RVEF, right ventricular ejection fraction.

**TABLE 5:** Cardiac magnetic resonance (CMR) tissue characterization.

Variable	All patients	Vaccine associated myocarditis	Non-vaccine associated myocarditis	*P*-value
**Myocardial injury localization**				
Anterior [*n* (%)]	4 (20)	1 (10)	3 (75)	0.264
Septal [*n* (%)]	1 (5)	0 (0)	1 (10)	0.305
Lateral [*n* (%)]	20 (100)	10 (100)	10 (100)	
Inferior [*n* (%)]	6 (30)	4 (40)	2 (20)	0.329
LGE presence [*n* (%)]	20 (100)	10 (100)	10 (100)	
Subendocardial [*n* (%)]	0 (0)	0 (0)	0 (0)	
Mid-wall [*n* (%)]	5 (25)	2 (20)	3 (30)	0.606
Subepicardial [*n* (%)]	20 (100)	10 (100)	10 (100)	
Transmural [*n* (%)]	0 (0)	0 (0)	0 (0)	
LGE (g)	5.3 (3.1–6.3)	4.4 (2.3–5.7)	6.0 (3.7–6.6)	0.089
LGE (%)	6.1 (4.6–7.0)	5.4 (3.7–6.7)	6.5 (5.2–7.9)	0.143
ECV global mean (%)	25.2 (23.5–28.4)	24.8 (23.3–26.7)	26.3 (23.5–29.9)	0.293
T1 native global mean (ms)	1,315 (1,276–1,349)	1,311 (1,282–1,342)	1,316 (1,261–1,369)	0.719
T1 post Gd global mean (ms)	502.6 (484.6–549.4)	506.6 (485.5–534.5)	496.8 (483.5–560.4)	0.797
T2 global mean (ms)	38.4 (36.1–39.7)	38.9 (35.8–39.8)	37.8 (36.2–39.19)	0.787
Maximum T1 native (ms)	1,625 (1,541–1,720)	1,618 (1,519–1,720)	1,633 (1,594–1,728)	0.684
High T1 native [*n* (%)]	20 (100%)	10 (100)	10 (100)	
Maximum T1 post Gd (ms)	581.0 (547.8–599.5)	582.0 (544.3–598.5)	575.0 (547.8–612.0)	0.912
Maximum T2 (ms)	53.0 (50.0–59.3)	52.0 (49.0–62.8)	54.5 (49.8–58.0)	0.796
High T2 [*n* (%)]	20 (100)	10 (100)	10 (100)	
Maximum T1 native/T1 native global mean	1.26 (1.16–1.31)	1.28 (1.14–1.31)	1.25 (1.20–1.31)	0.853
Maximum T1 post Gd/T1 post Gd global mean	1.1 (1.1–1.2)	1.1 (1.1–1.2)	1.1 (1.1–1.2)	0.796
Maximum T2/T2 global mean	1.39 (1.31–1.49)	1.35 (1.28–1.57)	1.39 (1.33–1.50)	0.724
Pericardial effusion	6 (30)	4 (40)	2 (20)	0.329

Data are presented as median (interquartile range), numbers and percentage. Comparisons of vaccine-associated myocarditis and non-vaccine-associated myocarditis were performed. Continuous parameters were tested for normal distribution using the Shapiro-Wilk test and compared using the Mann-Whitney U test or t-test as appropriate. Categorial parameters were tested using χ^2^ test. ULN: upper limit of normal; LGE: late gadolinium enhancement; ECV: extra cellular volume; Gd: gadolinium.

In all patients, LGE was present within the subepicardial layers without statistical differences regarding its relative enlargement within the myocardium (group 1: LGE 5.4%; group 2: LGE 6.5%; *p* = 0.143). Myocarditis affected the lateral segment in all cases, with partial involvement of the inferior segments in some of the patients (group 1: 40%; group 2: 20%; *p* = 0.329). A detailed overview is provided in Table 5.

One patient in each group showed artifacts within the anterior region of the myocardium in the T1 map. The affected segments were excluded from further analysis. Global T1 values were increased above the ULN for patients with both, vaccine-associated myocarditis and viral myocarditis (group 1: 1,311 ms; group 2: 1,316 ms). No significant differences in-between both groups could be observed (*p* = 0.719).

Segments with the highest T1 values were 23% above the global T1 in group 1 and 24% above the global T1 in group 2, respectively, without significant differences between both groups (*p* = 0.853).

**FIGURE 1:**
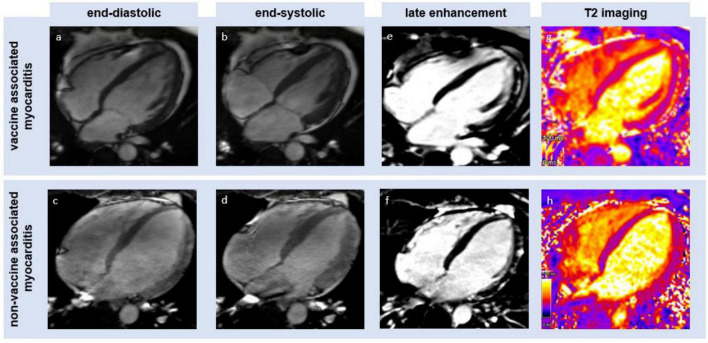
Illustration of cardiac magnetic resonance (CMR)-derived imaging (four chamber view) of an mRNA-based vaccination-associated myocarditis (upper row) and a non-vaccine-associated myocarditis (lower row). Panels **(a–d)** show left ventricular systolic function at the lower limit of normal. Panels **(e,f)** show a typical subepicardial late gadolinium enhancement. Panels **(g,h)** show a high signal on T2 mapping imaging as the result of edema.

Global T2 times were within normal ranges within both groups (group 1: 38.9 ms; group 2: 37.8 ms) with no significant differences (*p* = 0.787). Segmental T2 values at their maximum were numerically but not significantly higher within patients with viral myocarditis when compared to patients suffering from vaccine-associated myocarditis (group 1: 52.0 ms; group 2: 54.5 ms; *p* = 0.796). The latter values were 34% above the global T2 times in group 1 and 45% in group 2. Segments with the maximum T2 values were above the reference range in both groups. ECV was within the normal range in both groups (group 1: 24.8%; group 2: 26.3%; *p* = 0.293). An illustration of typical CMR findings for both groups is presented in Figure 1.

### Further diagnostic workup

In total, four patients underwent a CT scan to rule out a pulmonary embolism (group 1: three patients; group 2: one patient; *p* = 0.264) and in seven patients, invasive coronary angiography was performed (group 1: four patients; group 2: three patients; *p* = 0.639).

### Follow-up at discharge

In both groups, myocarditis-related symptoms, such as chest pain, were improved (*n* = 2; 10%) or even resolved (*n* = 18; 90%) at the time of discharge. The mean time of the hospital stay was 5 [3.8–6.3] days in group 1 when compared to 6 [4.8–7.0] days in group 2 (*p* = 0.653). Patients of both groups required non-steroidal anti-inflammatory drugs (group 1: 50%; group 2: 60%; *p* = 0.653) in equal partitions.

## Discussion

The main findings of the study are summarized by the following points:

1. COVID-19 vaccine-associated myocarditis and regular viral myocarditis share the same CMR patterns of inflammation during the acute phase in a small, carefully matched cohort of young male patients, representing the most affected patient group.
2. In particular, the CMR measurements did not reveal any differences in terms of morphological and functional data when compared to the matched control group suffering from regular viral myocarditis. This is in concordance with a similar clinical presentation, ECG changes, and assimilable echocardiographic findings in patients of both groups.
3. Late gadolinium enhancement was the predominant pathological marker of myocarditis in this study. No differences in the spatial arrangement of the affected regions were found and the underlying tissue differentiation using T1, T2, and ECV mapping techniques showed highly comparable results.
4. The observed increased T1 times rather seem to reflect a state of an acute inflammation than myocardial fibrosis, considering normal ECV values. In fact, both groups of patients showed focal edema pertaining to the inflamed area suggested by increased T2 values.

Since some evidence suggests distinct differences between vaccination-associated myocarditis and cardiac COVID-19-related involvement (so-called COVID-19 myocarditis), we now add further data on comparing vaccination-associated myocarditis and classical myocarditis. In opposite to COVID-19-related cardiac injury, our data suggest that vaccine-associated myocarditis and regular viral myocarditis show the same CMR patterns of inflammation (15).

In addition to comparable functional and morphological parameters in CMR, both groups had a similar clinical presentation. In our cohort, all patients suffered from chest pain as the main symptom, which was previously described in other populations with COVID-19 vaccination-related myocarditis, already (4–17).

Both types of myocarditis involved an equivalent amount of myocardium within the inflammation and in keeping with findings in other causes of myocarditis, COVID-19 vaccination-related myocarditis predominantly affected the subepicardial layers of the lateral wall of the left ventricle (17–19).

As increased T2 value within the inflamed areas might demarcate small focal edema, the global T2 values were below the upper threshold of abnormality, which suggests that no global edema was present in this very acute state. These observations agree with established knowledge of the progression of regular myocarditis and can be associated with minor myocardial damage.

Interestingly, imaging patterns of the viral and non-viral forms of myocarditis share the lateral wall as a predominant area of demarcation regardless of their distinct pathophysiology. This agrees with previous studies on both, viral myocarditis and COVID-19 vaccination-related myocarditis (31). In contrast to this, viral COVID-19 myocarditis was observed to show more diffusely distributed inflammation within the myocardium or a non-typical demarcation at the right ventricular insertion point (15–31). The pattern of lateral damage even in non-infectious causes, such as vaccination-related myocarditis, indicates a common ground lying pathophysiology, which may be related to immunologic reactions, which should be further investigated in future basic and translational research.

The similarity between the patterns of the vaccine-associated myocarditis and the regular viral myocarditis might be a reason for the fact that our control group was mostly balanced by laboratory markers, such as CK and troponin. Even if median troponin and CK levels in group 1 were higher as compared to group 2 without reaching statistical significance, both had analogical upper CK levels, which might suggest a comparable myocardial damage in both groups. However, it remains unclear, if the vaccine-associated myocarditis shows a similar progression as compared to other forms of myocarditis in general. Potentially, specific differences would have been shown up if fulminant forms of myocarditis would have been included. However, our findings agree with previously published data (4–32).

While the diagnosis of acute myocarditis is based on various parameters using T1- and T2-based imaging techniques (19), however, LGE, in particular, has been shown to be an important marker for risk stratification in non-ischemic cardiac myopathy (33). Myocardial deformation imaging, such as feature tracking or strain encoded (SENC) imaging, may provide additional capabilities for prognostication in non-ischemic cardiomyopathy and other patterns of myocardial injury (34–38), while being able to identify late-gadolinium-enhanced myocardial layers and even viable areas outside the directly affected regions (39–41). Furthermore, tissue tracking showed good agreement with ECV maps for the detection of myocardial fibrosis (42, 43). This offers a potential non-contrast-dependent diagnostic tool for tissue differentiation in the future. However, as different deformation imaging methods had a significantly varying agreement between the distinct techniques, global strain measurements showed the best reproducibility within each method (44). Therefore, we decided to just report global strain values for our study group, while the variability in-between the different techniques must be considered for interstudy comparisons and follow-ups.

In our matched study cohort, no differences could be observed with regard to volumetric and functional data on both echo and CMR. In contrast to this, a study by Fronza et al. recently described differences in functional parameters, such as GLS and LVEF, between COVID-19 vaccine-associated myocarditis and myocarditis of other causes with a trend of impaired left ventricular function in the non-vaccination-associated myocarditis group (17). As various aspects might impact this mismatch, it must be considered that the other study cohort was more heterogeneous, including women and older people, and CMR imaging was performed at a later timepoint after symptom onset. The combination of those factors might be a reason for the observed differences. In both studies, the presence of LGE was the parameter to majorly define the pathological presence of myocarditis and is in line with smaller case series (13).

Arguably, however, further differences might occur during later stages of myocarditis potentially offering a detailed insight into specific discrepancies of both forms of myocarditis and must be addressed in future prospective trials.

Notably, all patients with COVID-19 vaccination-related myocarditis have been found to be free of symptoms at the point of discharge already. While this observation is implying a promising outcome of vaccine-related myocarditis in young male patients as shown in earlier studies (10–13), a fast hospitalization and treatment after diagnosis might have been crucial to those results in our cohort.

Even though, we could not observe any adverse outcomes in our study cohort of young male patients with acute vaccine-associated myocarditis, this finding is limited by the small sample size. However, little is known about long-term follow-up data in patients suffering from mRNA vaccination-associated myocarditis. While in some cases, no pathological CMR patterns (increased T1 time and reduced LVEF) were resolved in the follow-up scan (45), other patients showed persistent LGE, even though initially abnormal global T1 times normalized and ECV values were decreased (18–46). As CMR shows promising capabilities for risk stratification in myocarditis, those preliminary results encourage future outcome studies, such as larger patient groups (47).

## Limitations

It must be taken into account that the study cohort was small and retrospectively matched. A subsequent selection bias cannot be fully excluded due to this study design. It should be part of future research work to sample a comprehensive cohort of all vaccinated people minimizing these limitations.

We have focused on the most affected group in the early stage of rare COVID-19 vaccine-associated myocarditis in a small number of patients. Therefore, our findings might not apply to the general population or other groups within vaccinated patients.

As exams at a later point of the disease’s progression might detect further specific patterns of myocarditis, our collective was sampled at an acute point after symptom onset. This agrees with the current guidelines, however, a prospective trial with follow-up surveys is highly desirable to address this (48).

Mapping was performed using only one midventricular short-axis slice. Therefore, any inflammation or fibrosis in more basal or apical segments could have been missed. However, measurements were performed equally in both patient groups and in accordance with available published literature on this research topic (17).

Finally, even if we could not find any evidence of an infection, an ischemic or autoimmune disease, the association of the myocarditis with the vaccination cannot be proved with absolute certainty. It remains a diagnosis by exclusion.

## Conclusion

COVID-19 mRNA vaccine-associated myocarditis does not show specific CMR patterns during the very acute stage in the most affected patient group of young men. The observed imaging findings are closely related to regular viral myocarditis and did not yield any evidence implying adverse outcomes in the investigated patient group.

## Data availability statement

All relevant data are within the article and all data underlying the findings are fully available without restriction and can be accessed at the University Medical Center Göttingen by researchers who meet the criteria for access to confidential data.

## Ethics statement

The studies involving human participants were reviewed and approved by Local Ethics Committee of the University Medical Center Göttingen. Written informed consent for participation was not required for this study in accordance with the national legislation and the institutional requirements.

## Author contributions

RE, AlS, and AnS: conceptualization, investigation, and writing – original draft preparation. RE and AlS: formal analysis. RE, AlS, TL, SB, DV, JK, SH, GH, and AnS: methodology, writing – review and editing, read, and agreed to the published version of the manuscript.
